# The Surgical Approach Combined With Minimally Invasive Surgery for Sacral Chordoma

**DOI:** 10.7759/cureus.18690

**Published:** 2021-10-11

**Authors:** Mauricio Garcia Mora, Ivan Fernando Mariño, Leidy Juliana Puerto Horta, Felipe Gonzalez, Sandra Diaz Casas

**Affiliations:** 1 Breast and Soft Tissue Surgery, Instituto Nacional de Cancerología, Bogotá D.C., COL; 2 Breast and Soft Tissue Surgery, Instituto Nacional de Cancerología, Bogota D.C., COL

**Keywords:** sacral chordoma, laparoscopy, sarcoma, surgical oncology, radiotherapy

## Abstract

Sacral chordomas are malignant, locally aggressive, and rare tumors. Its presentation can be diverse on the entire spine, being more frequent in the sacrococcygeal region. The main treatment is complete surgical resection, which can be performed using different approaches depending on the case. We present the case of a 44-year-old woman with a history of a complex adnexal mass, with an imaging finding of a presacral mass displacing the uterus and rectum, with a histological report of an image-guided biopsy suggestive of a soft-tissue myoepithelioma-like tumor, which was managed with a combined approach: anterior transabdominal laparoscopic and posterior approach, achieving complete tumor resection, without postoperative complications and with the benefits of minimally invasive surgery. The pathology report of the surgical piece was compatible with sacral chordoma.

## Introduction

Sacral chordomas are malignant, locally aggressive, and slow-growing tumors, originating in a notochord remnant [1]. They were first described by Virchow in 1857. They can affect any part of the neuraxis; although they are rare, they are the most frequent tumors of the sacrum and are more frequent in men than in women [2].

Its incidence varies between 0.18 and 0.84 per one million inhabitants per year [3]. In Colombia, the statistical yearbook of the National Cancer Institute of Colombia reported three new cases in 2018 [4]. They are usually asymptomatic or have non-specific symptoms related to compression or involvement of adjacent structures, which may delay diagnosis [5]. Recurrence and five-year overall survival rates of 63.1% and 41% have been reported, respectively [6].

Complete surgical resection of the sacral chordoma is the main treatment of both primary and recurrent localized disease [7]. The surgical management of the tumor is performed using three different approaches: anterior transabdominal, posterior, and combined. Tumors located below S3 can be resected with the posterior approach, while those located above this level can be managed using the anterior transabdominal route or the combined approach [8].

Currently, there are reports of cases that use a combined and laparoscopic approach, achieving complete tumor resection with lower morbidity [9].

No clinical benefits have been reported with the use of chemotherapy. Radiotherapy has been described in the neoadjuvant or adjuvant setting combined with surgical resection or as a primary treatment for patients with unresectable disease, or those who cannot tolerate surgery [10].

Next, we report the first case in our institution and the country of a combined transabdominal laparoscopic approach and a posterior approach with sacrectomy in a patient diagnosed with sacral chordoma.

## Case presentation

**Figure 1:**
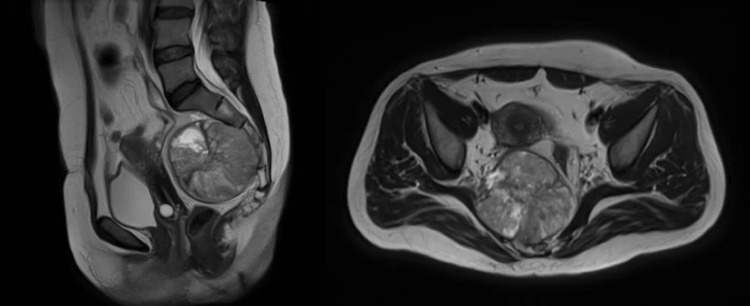
Magnetic resonance imaging. (A) Axial view and (B) sagittal view.

A 44-year-old woman with a history of a complex adnexal mass previously treated in another institution attends for the first time the Functional Unit for Breast and Soft Tissue Tumors due to the presence of a retro-uterine mass. The review by systems evidenced the presence of constipation of eight months of evolution, with identifying other symptoms or clinical signs. Magnetic resonance imaging of the pelvis showed a mass of 69 × 76 × 86 mm^3^ with its epicenter in the presacral fat, displacing the uterus and rectum, and infiltrating the S2, S3, and S4 vertebral bodies (Figure 1).

A thick needle biopsy (Core biopsy: TRU-CUT®) was performed guided by computed axial tomography. The histological study reported fragments of dense fibroconnective tissue with groups of epithelioid-like cells, broad eosinophilic cytoplasm, and slightly atypical/hyperchromatic central nucleus; cells arranged in nests with a slight overlap, some with the presence of nucleolus and focal stromal myxoid change, which are findings compatible with a soft-tissue myoepithelioma-like tumor (parachordoma). The patient was evaluated by a multidisciplinary board with the participation of oncologist surgeons, soft-tissue tumor surgeons, neurosurgeons, clinical oncologists, radiation oncologists, oncologic pathologists, and radiologists, establishing a treatment surgical approach combined with neurosurgery service.

Surgical technique

**Figure 2:**
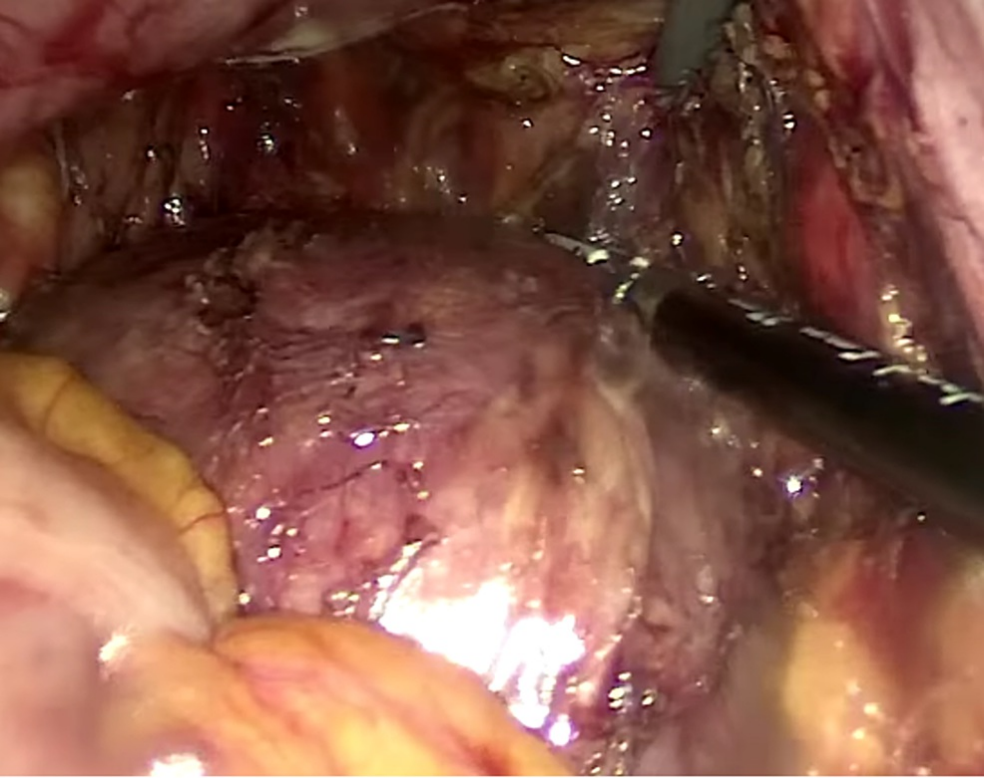
Laparoscopic view of resection.

**Figure 3:**
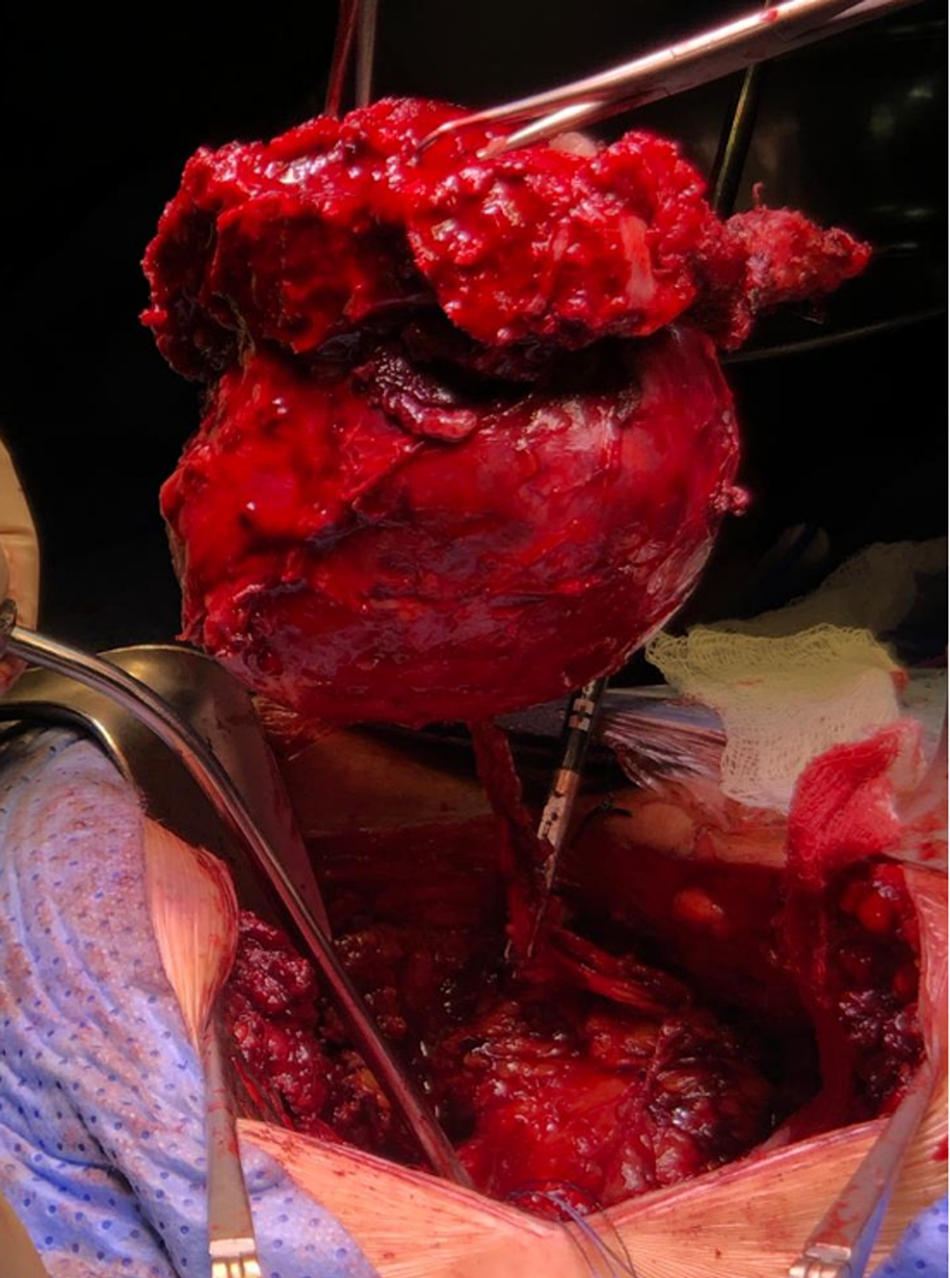
Bloc resection of sacral chordoma and distal segment of the sacrum.

In supine decubitus, under general anesthesia, an umbilical incision was made by open technique; the procedure consisted of the insertion of a 10-mm laparoscopic trocar, insufflation of the pneumoperitoneum, and insertion under direct vision of a 10-mm trocar in the right flank and a 5-mm trocar in the left iliac fossa and the right iliac fossa. The uterus and adnexa were tractioned anteriorly for better exposure. Dissection was initiated by mobilizing the sigmoid colon until entering the presacral space. Right and left ureters were identified with no evidence of involvement of the venous or arterial iliac vessels, or the pelvic walls. The avascular plane was identified; entering the presacral space, mesorectal dissection was performed exposing the tumor lesion (Figure 2), freeing it from lateral adhesions while it remained attached to the sacrum. Once the rectum was dissected and the tumor exposed, the following steps were performed: protection of the rectum, revision of hemostasis, removal of pneumoperitoneum, and closure of laparoscopic ports with suturing, in order to give way to the posterior approach by the neurosurgery group. In prone decubitus position, an incision was made in the lumbosacral region through the midline, followed by dissection and exposure of the sacral-coccygeal midline and bilateral sacroiliac joint, laminectomy and identification of low roots, osteotomies at the S2 level and sacroiliac joints, preserving the roots of S2 while achieving complete en bloc removal of the lesion and the sacrum (Figure 3). The posterior wall was reconstructed with tissue-separating mesh and suture by planes.

In the immediate postoperative period, the patient was monitored in the intensive care unit for two days and in a general hospital room for four additional days, for a total hospital stay of six days with satisfactory evolution.

The pathological study of the surgical piece reported compromise due to chordoma-type notochordal neoplasia with negative resection margins for tumor involvement. After five months, the patient does not present sensory or motor deficits, she has adequate sphincter control, and there is no evidence of relapse on control images.

## Discussion

The treatment of sacral chordoma is a surgical challenge, due to its location, size, infrequent presentation, and histological findings difficult to classify, which pose the need for a differential diagnosis from chondrosarcoma, plasmacytoma, giant cell tumor, or metastatic disease that may require a different therapeutic approach; thus, it must be treated in reference centers and by multidisciplinary groups [6].

Complete tumor resection is the main prognostic factor, with an impact on overall survival and disease-free survival, which is why adequate imaging studies are required for its planning, including magnetic resonance imaging or computerized axial tomography. The appropriate surgical approach is recommended according to tumor location and size; thus, for small and localized tumors below S3, the posterior approach is proposed, while for tumors located above S3 a combined or anterior transabdominal approach is suggested, depending on the need for bone resection and the experience of the surgeon.

Thanks to the experience gained from laparoscopic colon and rectal resections, the high anatomical knowledge and multidisciplinary work of the institution, as well as the characteristics of the tumor; the laparoscopic approach allowed an adequate visualization of the structures and magnification of the resection, which is difficult to achieve with a traditional open surgical approach. Additionally, it has been associated with lower morbidity, less postoperative pain, shorter hospital stay, and improved quality of life [11,12].

## Conclusions

When a tumor occurs in the sacrum, it is important to perform an adequate histological classification; thus, the patient must be referred to an oncological institution that has experience in the management of these types of tumors. The laparoscopic approach should be considered for tumor resection since it allows obtaining satisfactory results in relation to pain, morbidity, and hospital stay while ensuring adequate oncological margins.
